# Supplementary material for: Systematic Review: Contribution of the Gut Microbiome to the Volatile Metabolic Fingerprint of Colorectal Neoplasia
Source: Metabolites. 2022 Dec 30;13(1):55. doi: 10.3390/metabo13010055 (PMC9865897; doi:10.3390/metabo13010055)
Supplement: Supplementary file 1 [file metabolites-13-00055-s001.zip › metabolites-2087034-supplementary/Supplementary_Materials_S2_2022-02-11 web of science search VOCs.pdf]

English Products

Web of Science™ Search Marked List History Alerts Sign In Register

< BACK TO BASIC SEARCHES

Advanced Search Query Builder

DOCUMENTS RESEARCHERS

Search in: **Web of Science Core Collection** Editions: **All**

Add terms to the query search preview

All Fields | Example: liver disease india singh

And

Add to query

More options

Query Preview

ALL=(rectal neoplasm)

Add date range | Clear | Add to history

Search Help

Booleans : AND, OR, NOT

Field Tags :

- TS=Topic
- TI=Title
- AB=Abstract
- AU=[Author]
- AI=Author Identifiers
- AK=Author Keywords
- GP=[Group Author]
- ED=Editor
- KP=Keyword Plus ®
- SO=[Publication Titles]
- DO=DOI
- PY=Year Published
- CF=Conference
- AD=Address
- OG=[Affiliation]
- OO=Organization
- SG=Suborganization
- SA=Street Address
- CI=City
- PS=Province/State
- CU=Country/Region
- ZP=Zip/Postal Code
- FO=Funding Agency
- FG=Grant Number
- FD=Funding Details
- FT=Funding Text
- SU=Research Area
- WC=Web of Science Categories
- IS= ISSN/ISBN
- UT=Accession Number
- PMID=PubMed ID
- DOP=Publication Date
- PUBL=Publisher
- ALL=All Fields
- FPY=Final publication year

Session Queries

Build a new query based on your searches in this session.

35/35 Combine Sets Clear History

#	Query	Results	
35	#33 AND #34	1,107	Add to query
34	#6 OR #14	268,932	Add to query
33	#32 OR #31 OR #29 OR #30 OR #28 OR #27 OR #25 OR #26 OR #24 OR #23 OR #22 OR #21 OR #20	359,609	Add to query
32	ALL=(rectal neoplasm)	4,516	Add to query
31	ALL=(rectal neoplasms)	4,518	Add to query
30	ALL=(colonic neoplasms)	3,179	Add to query
29	ALL=(colonic neoplasm)	3,177	Add to query
28	ALL=(colorectal	13,190	Add to query

36

[illegible]	neoplasms)	[illegible]	Add to query
27	ALL=(colorectal neoplasm)	13,172	Add to query
26	ALL=(colorectal adenoma)	18,082	Add to query
25	ALL=(colorectal polyps)	14,669	Add to query
24	ALL=(colorectal polyp)	14,657	Add to query
23	ALL=(colorectal neoplasms)	13,190	Add to query
22	ALL=(colorectal neoplasm)	13,172	Add to query
21	ALL=(colorectal neoplasia)	10,323	Add to query
20	ALL=(colorectal tumor)	109,642	Add to query
19	ALL=(colorectal cancer)	241,300	Add to query
18	ALL=(rectal cancer)	55,833	Add to query
17	ALL=(colon cancer)	150,939	Add to query
16	ALL=(colorectal carcinoma)	69,007	Add to query
15	ALL=(colorectal cancer)	241,300	Add to query
14	#8 OR #13 OR #11 OR #10 OR #12 OR #9 OR #7	229,363	Add to query
13	ALL=(Selected-ion flow-tube)	915	Add to query
12	ALL=(GCxGC)	1,247	Add to query
11	ALL=(GCMS)	9,047	Add to query
10	ALL=(GC MS)	113,080	Add to query
9	ALL=(Gas chromatography mass spectrometry)	86,440	Add to query
8	ALL=(Gas chromatography mass spectrometry)	86,440	Add to query

36

7	ALL=(Gas chromatography)	154,211	Add to query
6	#5 OR #4 OR #3 OR #2 OR #1	50,163	Add to query
5	ALL=(breathomics)	109	Add to query
4	ALL=(volatolomics)	68	Add to query
3	ALL=(volatolome)	22	Add to query
2	ALL=(volatile organic compounds)	50,096	Add to query
1	ALL=(volatile organic compound)	49,888	Add to query

Netherlands Consortium Maastricht University

© 2021 Clarivate
Training Portal
Product Support
Data Correction
Privacy Statement
Newsletter
Copyright Notice
Cookie Policy
Terms of Use
Cookie Settings
Follow Us

36
