# Supplementary material for: Systematic Review: Contribution of the Gut Microbiome to the Volatile Metabolic Fingerprint of Colorectal Neoplasia
Source: Metabolites. 2022 Dec 30;13(1):55. doi: 10.3390/metabo13010055 (PMC9865897; doi:10.3390/metabo13010055)
Supplement: Supplementary file 1 [file metabolites-13-00055-s001.zip › metabolites-2087034-supplementary/Supplementary_Materials_S3_2022-02-18 web of science search.pdf]

English Products

Web of Science™ Search Marked List History Alerts Sign In Register

< BACK TO BASIC SEARCHES

Advanced Search Query Builder

DOCUMENTS RESEARCHERS

Search in: **Web of Science Core Collection** Editions: **All**

Add terms to the query search preview

All Fields | Example: liver disease india singh

And

Add to query

More options

Query Preview

ALL=(metagenome)

+ Add date range | Clear | Add to history

Search Help

Booleans : AND, OR, NOT

Field Tags :

- TS=Topic
- TI=Title
- AB=Abstract
- AU=[Author]
- AI=Author Identifiers
- AK=Author Keywords
- GP=[Group Author]
- ED=Editor
- KP=Keyword Plus ®
- SO=[Publication Titles]
- DO=DOI
- PY=Year Published
- CF=Conference
- AD=Address
- OG=[Affiliation]
- OO=Organization
- SG=Suborganization
- SA=Street Address
- CI=City
- PS=Province/State
- CU=Country/Region
- ZP=Zip/Postal Code
- FO=Funding Agency
- FG=Grant Number
- FD=Funding Details
- FT=Funding Text
- SU=Research Area
- WC=Web of Science Categories
- IS= ISSN/ISBN
- UT=Accession Number
- PMID=PubMed ID
- DOP=Publication Date
- PUBL=Publisher
- ALL=All Fields
- FPY=Final publication year

Session Queries

Build a new query based on your searches in this session.

1/41 Combine Sets Clear History

#	Query	Results	
41	**#29 AND #19** and **Meta Analysis** (Search within all fields)	87	Add to query
40	**#29 AND #19** and **Meta-analysis** (Search within all fields)	76	Add to query
39	**#29 AND #19** and **Meta Analysis** (Search within all fields)	87	Add to query
38	**#29 AND #19** and **Systematic Review** (Search within all fields)	98	Add to query
37	**#29 AND #19** and **Review Articles** (Document Types) and **Systematic Review** (Search within all [illegible]	86	Add to query

36

☐	36	**#29 AND #19** and **Review Articles** (Document Types) and **Review Articles** (Document Types) and	86	Add to query
☐	35	**#29 AND #19** and **Review Articles** (Document Types) and **Review Articles** (Document Types) and	86	Add to query
☐	34	**#29 AND #19** and **Review Articles** (Document Types) and **Review Articles** (Document Types) and	35	Add to query
☐	33	**#29 AND #19** and **Review Articles** (Document Types) and **Review Articles** (Document Types) and	86	Add to query
☐	32	**#29 AND #19** and **Review Articles** (Document Types) and **Review Articles** (Document Types)	1,380	Add to query
☐	31	**#29 AND #19** and **Review Articles** (Document Types)	1,380	Add to query
☐	30	**#29 AND #19**	4,623	Add to query
☐	29	**#20 OR #21 OR #22 OR #23 OR #24 OR #25 OR #26 OR #27 OR #28**	140,227	Add to query
☐	28	**ALL=(metagenome)**	6,329	Add to query
☐	27	**ALL=(metagenomics)**	11,540	Add to query
☐	26	**ALL=(mycobiota)**	1,712	Add to query
☐	25	**ALL=(mycobiome)**	796	Add to query
☐	24	**ALL=(microbiota)**	96,187	Add to query
☐	23	**ALL=(gut microbiota)**	57,145	Add to query
☐	22	**ALL=(gut microbiome)**	27,468	Add to query
☐	21	**ALL=(gastrointestinal microbiome)**	6,442	Add to query
☐	20	**ALL=(microbiome)**	57,724	Add to query

36

19	#18 OR #17 OR #16 OR #15 OR #14 OR #13 OR #12 OR #11 OR #10 OR #9 OR #8 OR #7 OR #6 OR #5	359,907	Add to query
18	ALL=(rectal neoplasm)	4,523	Add to query
17	ALL=(rectal neoplasms)	4,525	Add to query
16	ALL=(colonic neoplasms)	3,185	Add to query
15	ALL=(colonic neoplasm)	3,183	Add to query
14	ALL=(colorectal neoplasms)	13,210	Add to query
13	ALL=(colorectal neoplasm)	13,192	Add to query
12	ALL=(colorectal adenoma)	18,097	Add to query
11	ALL=(colorectal polyps)	14,683	Add to query
10	ALL=(colorectal polyp)	14,671	Add to query
9	ALL=(colorectal neoplasms)	13,210	Add to query
8	ALL=(colorectal neoplasm)	13,192	Add to query
7	ALL=(colorectal neoplasia)	10,333	Add to query
6	ALL=(colorectal tumor)	109,762	Add to query
5	ALL=(colorectal cancer)	241,539	Add to query
4	ALL=(rectal cancer)	55,873	Add to query
3	ALL=(colon cancer)	151,056	Add to query
2	ALL=(colorectal carcinoma)	69,063	Add to query
1	ALL=(colorectal cancer)	241,539	Add to query

Netherlands Consortium Maastricht University

36

© 2021 Clarivate
Training Portal
Product Support
Data Correction
Privacy Statement
Newsletter
Copyright Notice
Cookie Policy
Terms of Use
Cookie Settings
Follow Us

36
