# Supplementary material for: Systematic Review: Contribution of the Gut Microbiome to the Volatile Metabolic Fingerprint of Colorectal Neoplasia
Source: Metabolites. 2022 Dec 30;13(1):55. doi: 10.3390/metabo13010055 (PMC9865897; doi:10.3390/metabo13010055)
Supplement: Supplementary file 1 [file metabolites-13-00055-s001.zip › metabolites-2087034-supplementary/Supplementary_Materials_S4_Included_VOC_Studies.pdf]

Supplementary materials S4 – Included studies

Table S1. Summary of studies analyzing fecal VOCs for the detection of colorectal neoplasia compared to controls.

Author	Comparison	Samples	Sampling method	Analysis method	Microbiota analysis	Reference
Chen *et al.* 2013	AA vs healthy	344 CRC, 344 healthy	Derivatization	GC-MS	Yes	[33]
Weir *et al.* 2013	CRC vs healthy	11 CRC, 10 healthy	Derivatization	GC-MS	Yes	[87]
Phua *et al*. 2014	CRC vs healthy	11 CRC, 10 healthy	Derivatization	GC-MS	No	[71]*
Batty *et al.* 2015	CRC+AA vs healthy	31 CRC+AA, 31 healthy	Nalophan	SIFT-MS	No	[98]
Bond *et al.* 2019	CRC vs healthy	21 CRC, 60 control	SPME	GC-MS	No	[31]
Ishibe *et al.* 2018	CRC vs healthy	22 CRC, 26 control	Tedlar	GC-MS	No	[49]
Niccolai *et al.* 2019	CRC vs healthy	19 CRC, 16 control	Derivatization	GC-MS	No	[63]
Yachida *et al.* 2019	CRC vs healthy	178 CRC, 149 healthy	Derivatization	CE-*tof*-MS	Yes	[89]
Yang *et al.* 2019	CRC vs healthy	50 CRC, 50 healthy	Derivatization	GC-MS	Yes	[90]
Yusuf *et al.* 2019	CRC vs healthy	14 CRC, 14 healthy	Derivatization	GC-MS	Yes	[92]
Kim *et al.* 2020	CRC vs healthy	32 CRC, 44 healthy	Derivatization	GC-MS	Yes	[52]
Coker *et al.* 2022	CRC vs AA vs healthy	118 CRC, 140 AA, 128 healthy	Derivatization	GC-MS	Yes	[36]

CRC: colorectal carcinoma, AA: advanced adenoma, GC-MS: gas chromatography-mass spectrometry, CE-tof-MS: capillary electrophoresis-time of flight-mass spectrometry, SIFT-MS: selected ion flow tube-mass spectrometry, SPME: solid phase microextraction

* also analyzed VOCs in tissue

References

31 Bond, A.; Greenwood, R.; Lewis, S.; Corfe, B.; Sarkar, S.; O'Toole, P.; Rooney, P.; Burkitt, M.; Hold, G.; Probert, C. Volatile organic compounds emitted from faeces as a biomarker for colorectal cancer. *Aliment. Pharmacol. Ther*. **2019**, *49*, 1005–1012. https://doi.org/10.1111/apt.15140.

33 Chen, H.M.; Yu, Y.N.; Wang, J.L.; Lin, Y.W.; Kong, X.; Yang, C.Q.; Yang, L.; Liu, Z.J.; Yuan, Y.Z.; Liu, F.; et al. Decreased dietary fiber intake and structural alteration of gut microbiota in patients with advanced colorectal adenoma. *Am. J. Clin. Nutr*. **2013**, *97*, 1044–1052. https://doi.org/10.3945/ajcn.112.046607.

36 Coker, O.O.; Liu, C.; Wu, W.K.K.; Wong, S.H.; Jia, W.; Sung, J.J.Y.; Yu, J. Altered gut metabolites and microbiota interactions are implicated in colorectal carcinogenesis and can be non-invasive diagnostic biomarkers. *Microbiome* **2022**, *10*, 35. https://doi.org/10.1186/s40168-021-01208-5.

49 Ishibe, A.; Ota, M.; Takeshita, A.; Tsuboi, H.; Kizuka, S.; Oka, H.; Suwa, Y.; Suzuki, S.; Nakagawa, K.; Suwa, H.; et al. Detection of gas components as a novel diagnostic method for colorectal cancer. *Ann. Gastroenterol. Surg*. **2018**, *2*, 147–153. https://doi.org/10.1002/ags3.12056.

52 Kim, D.J.; Yang, J.; Seo, H.; Lee, W.H.; Lee, D.H.; Kym, S.; Park, Y.S.; Kim, J.G.; Jang, I.-J.; Kim, Y.-K.; et al. Colorectal cancer diagnostic model utilizing metagenomic and metabolomic data of stool microbial extracellular vesicles. *Sci. Rep*. **2020**, *10*, 2860. https://doi.org/10.1038/s41598-020-59529-8.

63 Niccolai, E.; Baldi, S.; Ricci, F.; Russo, E.; Nannini, G.; Menicatti, M.; Poli, G.; Taddei, A.; Bartolucci, G.; Calabrò, A.S.; et al. Evaluation and comparison of short chain fatty acids composition in gut diseases. *World J. Gastroenterol*. **2019**, *25*, 5543–5558. https://doi.org/10.3748/wjg.v25.i36.5543.

71 Phua, L.C.; Chue, X.P.; Koh, P.K.; Cheah, P.Y.; Ho, H.K.; Chan, E.C.Y. Non-invasive fecal metabonomic detection of colorectal cancer. *Cancer Biol. Ther.* **2014**, *15*, 389–397. https://doi.org/10.4161/cbt.27625.

87 Weir, T.L.; Manter, D.K.; Sheflin, A.M.; Barnett, B.A.; Heuberger, A.L.; Ryan, E.P. Stool Microbiome and Metabolome Differences between Colorectal Cancer Patients and Healthy Adults. *PLoS ONE* **2013**, *8*, e70803. https://doi.org/10.1371/journal.pone.0070803.

89 Yachida, S.; Mizutani, S.; Shiroma, H.; Shiba, S.; Nakajima, T.; Sakamoto, T.; Watanabe, H.; Masuda, K.; Nishimoto, Y.; Kubo, M.; et al. Metagenomic and metabolomic analyses reveal distinct stage-specific phenotypes of the gut microbiota in colorectal cancer. *Nat. Med*. **2019**, *25*, 968–976. https://doi.org/10.1038/s41591-019-0458-7.

90 Yang, Y.; Misra, B.; Liang, L.; Bi, D.; Weng, W.; Wu, W.; Cai, S.; Qin, H.; Goel, A.; Li, X.; et al. Integrated microbiome and metabolome analysis reveals a novel interplay between commensal bacteria and metabolites in colorectal cancer. *Theranostics* **2019**, *9*, 4101–4114. https://doi.org/10.7150/thno.35186.

92 Yusuf, F.; Adewiah, S.; Syam, A.F.; Fatchiyah, F. Altered profile of gut microbiota and the level short chain fatty acids in colorectal cancer patients. *J. Phys. Conf. Ser*. **2019**, *1146*, 012037. https://doi.org/10.1088/1742-6596/1146/1/012037.

98 Batty, C.A.; Cauchi, M.; Lourenço, C.; Hunter, J.O.; Turner, C. Use of the analysis of the volatile faecal metabolome in screening for colorectal cancer. *PLoS ONE* **2015**, *10*, e0130301. https://doi.org/10.1371/journal.pone.0130301.

Table S2. Summary of studies analyzing urinary VOCs for the detection of colorectal neoplasia compared to controls.

Author	Comparison	Samples	Sampling method	Analysis method	Microbiota analysis	Reference
Qiu *et al.* 2010	CRC vs healthy	60 CRC, 63 healthy	Derivatization	GC-MS	No	[75]
Silva *et al.* 2011	CRC vs healthy (vs leukemia vs lymphoma)	11 CRC, 21 healthy	Dynamic SPME	GC-MS	No	[80]
Chen *et al.* 2012	CRC vs healthy	20 CRC, 14 healthy	Derivatization	CE-MS	No	[34]
Cheng *et al.* 2012	CRC vs healthy	101 CRC, 103 healthy	Derivatization	GC-MS	No	[35]
Arasaradnam *et al.* 2014	CRC vs healthy	83 CRC, 50 healthy	Direct headspace	GC-MS	No	[62]
Rozhentsov *et al.* 2014	CRC vs healthy (vs gastric carcinoma vs lung carcinoma)	14 CRC, 35 healthy	SPME	GC-MS	No	[78]
Porto-Figueira *et al.* 2018	CRC vs healthy	30 CRC, 30 healthy	SPME	GC-MS	No	[74]
Ning *et al.* 2021	CRC vs healthy	163 CRC, 111 healthy	Derivatization	GC-MS	No	[65]
Tyagi *et al.* 2021	CRC vs healthy	58 CRC, 38 healthy	TD headspace	GC-MS	No	[82]

CRC: colorectal carcinoma, GC-MS: gas chromatography-mass spectrometry, SPME: solid phase microextraction, CE-MS: capillary electrophoresis-mass spectrometry, TD: thermal desorption.

References

34 Chen, J.L.; Fan, J.; Yan, L.S.; Guo, H.Q.; Xiong, J.J.; Ren, Y.; Hu, J.D. Urine metabolite profiling of human colorectal cancer by capillary electrophoresis mass spectrometry based on MRB. *Gastroenterol. Res. Pract*. **2012**, *2012*, 125890. https://doi.org/10.1155/2012/125890.

35 Cheng, Y.; Xie, G.; Chen, T.; Qiu, Y.; Zou, X.; Zheng, M.; Tan, B.; Feng, B.; Dong, T.; He, P.; et al. Distinct urinary metabolic profile of human colorectal cancer. *J. Proteome Res*. **2012**, *11*, 1354-1363. https://doi.org/10.1021/pr201001a.

62 Arasaradnam, R.P.; McFarlane, M.J.; Ryan-Fisher, C.; Westenbrink, E.; Hodges, P.; Thomas, M.G.; Chambers, S.; O'Connell, N.; Bailey, C.; Harmston, C.; et al. Detection of colorectal cancer (CRC) by urinary volatile organic compound analysis. *PLoS ONE* **2014**, *9*, e108750. https://doi.org/10.1371/journal.pone.0108750.

65 Ning, W.; Qiao, N.; Zhang, X.; Pei, D.; Wang, W. Metabolic profiling analysis for clinical urine of colorectal cancer. *Asia Pac. J. Clin. Oncol*. **2021**, *17*, 403–413. https://doi.org/10.1111/ajco.13591.

74 Porto-Figueira, P.; Pereira, J.A.M.; Câmara, J.S. Exploring the potential of needle trap microextraction combined with chromatographic and statistical data to discriminate different types of cancer based on urinary volatomic biosignature. *Anal. Chim. Acta*. **2018**, *1023*, 53–63. https://doi.org/10.1016/j.aca.2018.04.027.

75 Qiu, Y.; Cai, G.; Su, M.; Chen, T.; Liu, Y.; Xu, Y.; Ni, Y.; Zhao, A.; Cai, S.; Xu, L.X.; et al. Urinary metabonomic study on colorectal cancer. *J. Proteome. Res*. **2010**, *9*, 1627–1634. https://doi.org/10.1021/pr901081y.

78 Rozhentsov, A.A.; Koptina, A.V.; Mitrakov, N.N.; Sharipova, T.; Tsapaev, I.; Ryzhkov, V.L.; Lychagin, K.A.; Furina, R.R.; Mitrakova, N.N. A new method to diagnose cancer based on image analysis of mass chromatograms of volatile organic compounds in urine. *Sovrem. Tehnol. V Med*. **2014**, *6*, 151–157.

80 Silva, C.L.; Passos, M.; Cmara, J.S. Investigation of urinary volatile organic metabolites as potential cancer biomarkers by solid-phase microextraction in combination with gas chromatography-mass spectrometry. *Br. J. Cancer* **2011**, *105*, 1894–1904. https://doi.org/10.1038/bjc.2011.437.

82 Tyagi, H.; Daulton, E.; Bannaga, A.S.; Arasaradnam, R.P.; Covington, J.A. Non-invasive detection and staging of colorectal cancer using a portable electronic nose. *Sensors* **2021**, *21*, 5440. https://doi.org/10.3390/s21165440.

Table S3. Summary of studies analyzing breath VOCs for the detection of colorectal neoplasia compared to controls.

**Author**	**Comparison**	**Samples**	**Sampling method**	**Analysis method**	**Microbiota analysis**	**Reference**
Peng *et al.* 2010	CRC versus healthy	26 CRC, 22 controls	Mylar bag	GC-MS	No	[70]
Altomare *et al.* 2013	CRC vs healthy	37 CRC, 41 healthy	Tedlar	GC-MS	No	[29]
Wang *et al.* 2014	CRC vs healthy	20 CRC, 20 healthy	Syringe, SPME	GC-MS	No	[85]
Altomare *et al.* 2015	CRC vs previous CRC	32 CRC, 55 healthy	Tedlar	GC-MS	No	[40]
Amal *et al.* 2016	CRC vs healthy	65 CRC, 122 healthy	Tedlar	GC-MS	No	[51]
Markar *et al.* 2019	CRC versus GI disease vs healthy	50 CRC, 50 positive controls, 50 healthy	Nalophan	SIFT-MS	No	[60]
Altomare *et al.* 2020	Early CRC vs healthy	83 CRC, 90 healthy	ReCIVA	GC-MS	No	[28]
De Vietro *et al.* 2020	CRC vs healthy	7 CRC, 20 healthy	ReCIVA	GC-MS	No	[41]*
Miller-Atkins *et al.* 2020	CRC (liver metastases) vs HCC vs cirrhosis vs healthy	54 CRC, 51 healthy	Mylar bag	SIFT-MS	No	[61]
Politi *et al.* 2021	CRC vs lung adenocarcinoma vs lung squamous cell carcinoma vs healthy	52 CRC, 45 healthy	Bio-VOC sampler	IMR-MS	No	[72]
Cheng *et al.* 2022	CRC versus AA versus healthy	30 CRC, 138 AA, 130 NAA, 84 healthy	Tedlar	GC-MS	No	[96]

CRC: colorectal carcinoma, AA: advanced adenoma, NAA: non-advanced adenoma, GC-MS: gas chromatography-mass spectrometry, SPME: solid phase microextraction, SIFT-MS: selected ion flow tube-mass spectrometry, IMR-MS: ion molecule reaction-mass spectrometry

* also analyzed VOCs in tissue

References

28 Altomare, D.F.; Picciariello, A.; Rotelli, M.T.; De Fazio, M.; Aresta, A.; Zambonin, C.G.; Vincenti, L.; Trerotoli, P.; De Vietro, N. Chemical signature of colorectal cancer: Case-control study for profiling the breath print. *BJS Open* **2020**, *4*, 1189–1199. https://doi.org/10.1002/bjs5.50354.

29 Altomare, D.F.; Di Lena, M.; Porcelli, F.; Trizio, L.; Travaglio, E.; Tutino, M.; Dragonieri, S.; Memeo, V.; De Gennaro, G. Exhaled volatile organic compounds identify patients with colorectal cancer. *Br. J. Surg*. **2013**, *100*, 144–150. https://doi.org/10.1002/bjs.8942.

40 Altomare, D.F.; Di Lena, M.; Porcelli, F.; Travaglio, E.; Longobardi, F.; Tutino, M.; Depalma, N.; Tedesco, G.; Sardaro, A.; Memeo, R.; et al. Effects of curative colorectal cancer surgery on exhaled volatile organic compounds and potential implications in clinical follow-up. *Ann. Surg*. **2015**, *262*, 862–867. https://doi.org/10.1097/SLA.0000000000001471.

41 De Vietro, N.; Aresta, A.; Rotelli, M.T.; Zambonin, C.; Lippolis, C.; Picciariello, A.; Altomare, D.F. Relationship between cancer tissue derived and exhaled volatile organic compound from colorectal cancer patients. Preliminary results. *J. Pharm. Biomed. Anal*. **2020**, *180*, 113055. https://doi.org/10.1016/j.jpba.2019.113055.

51 Amal, H.; Leja, M.; Funka, K.; Lasina, I.; Skapars, R.; Sivins, A.; Ancans, G.; Kikuste, I.; Vanags, A.; Tolmanis, I.; et al. Breath testing as potential colorectal cancer screening tool. *Int. J. Cancer* **2016**, *138*, 229–236. https://doi.org/10.1002/ijc.29701.

60 Markar, S.R.; Chin, S.; Romano, A.; Wiggins, T.; Antonowicz, S.; Paraskeva, P.; Ziprin, P.; Darzi, A.; Hanna, G.B. Breath Volatile Organic Compound Profiling of Colorectal Cancer Using Selected Ion Flow-tube Mass Spectrometry. *Ann. Surg*. **2019**, *269*, 903–910. https://doi.org/10.1097/SLA.0000000000002539.

61 Miller-Atkins, G.; Acevedo-Moreno, L.-A.; Grove, D.; Dweik, R.A.; Tonelli, A.R.; Brown, J.M.; Allende, D.S.; Aucejo, F.; Rotroff, D.M. Breath Metabolomics Provides an Accurate and Noninvasive Approach for Screening Cirrhosis, Primary, and Secondary Liver Tumors. *Hepatol. Commun*. **2020**, *4*, 1041–1055. https://doi.org/10.1002/hep4.1499.

70 Peng, G.; Hakim, M.; Broza, Y.Y.; Billan, S.; Abdah-Bortnyak, R.; Kuten, A.; Tisch, U.; Haick, H. Detection of lung, breast, colorectal, and prostate cancers from exhaled breath using a single array of nanosensors. *Br. J. Cancer* **2010**, *103*, 542–551. https://doi.org/10.1038/sj.bjc.6605810.

72 Politi, L.; Monasta, L.; Rigressi, M.; Princivalle, A.; Gonfiotti, A.; Camiciottoli, G.; Perbellini, L. Discriminant profiles of volatile compounds in the alveolar air of patients with squamous cell lung cancer, lung adenocarcinoma or colon cancer. *Molecules* **2021**, *26*, 550. https://doi.org/10.3390/molecules26030550.

85 Wang, C.; Ke, C.; Wang, X.; Chi, C.; Guo, L.; Luo, S.; Guo, Z.; Xu, G.; Zhang, F.; Li, E. Noninvasive detection of colorectal cancer by analysis of exhaled breath. *Anal. Bioanal. Chem*. **2014**, *406*, 4757–4763. https://doi.org/10.1007/s00216-014-7865-x.
96 Cheng, H.R.; van Vorstenbosch, R.W.; Pachen, D.M.; Meulen, L.W.; Straathof, J.W.A.; Dallinga, J.W.; Jonkers, D.M.; Masclee, A.A.; van Schooten, F.-J.; Mujagic, Z.; et al. Detecting Colorectal Adenomas and Cancer Via Volatile Organic Compounds in Exhaled Breath, a Proof of Principle Study to Improve Screening. *Clin. Transl. Gastroenterol*. **2022**, *13*, e00518. https://doi.org/10.14309/ctg.0000000000000518.

Table S4. Summary of studies analyzing VOCs in blood for the detection of colorectal neoplasia compared to controls.

**Author**	**Comparison**	**Samples**	**Sampling method**	**Analysis method**	**Microbiota analysis**	**Reference**
Qiu *et al.* 2009	CRC vs healthy	54 CRC, 65 controls	Derivatization	GC-MS	No	[76]
Ma *et al.* 2010	Pre vs post CRC	30 pre-surgery, 30 post-surgery	Derivatization	GC-MS	No	[56]
Nishiumi *et al.* 2012	CRC vs healthy	40 CRC per stage, 40 controls	Derivatization	GC-MS	No	[66]
Ikeda *et al.* 2012	CRC vs healthy controls	12 CRC, 12 controls	Derivatization	GC-MS	No	[48]
Ma *et al.* 2012	CRC vs healthy	30 CRC, 30 controls	Derivatization	GC-MS	No	[57]
Tan *et al.* 2013	CRC vs healthy	101 CRC, 102 healthy	Derivatization	GC-MS	No	[81]
Wang et al .2014	CRC vs healthy	16 CRC, 20 healthy	SPME	GC-MS	No	[86]
Cross *et al.* 2014	CRC versus controls	254 CRC versus 254 controls	Derivatization	GC-MS	No	[37]
Crotti *et al.* 2016	CRC vs healthy	48 CRC, 20 controls	Derivatization	GC-MS	No	[38]
Farshidfar *et al.* 2016	CRC vs controls	320 CRC, 254 controls	Derivatization	GC-MS	No	[44]
Uchiyama *et al.* 2017	CRC vs healthy	56 CRC, 60 healthy	Derivatization	GC-MS	No	[83]
Nishiumi *et al.* 2017	CRC vs healthy	282 CRC, 291 healthy	Derivatization	GC-MS	No	[67]
Shu *et al.* 2018	CRC vs healthy	245 CRC, 245 control	Derivatization	GC-MS	No	[79]
Kim *et al.* 2019	CRC vs healthy	30 CRC, 30 controls	SPME	GCxGC-MS	No	[53]
Di Giovanni *et al.* 2020	CRC vs healthy	18 CRC, 19 healthy	Derivatization	GCxGC-MS	No	[43]
Hassan *et al.* 2020	CRC vs controls	32 CRC, 32 controls	Derivatization	GC-MS	No	[46]
Yuan *et al.* 2020	CRC vs healthy	29 CRC, 29 healthy	Derivatization	GCxGC-MS	No	[91]
Wu *et al.* 2020	CRC vs healthy	22 CRC, 23 RC, 45 healthy	Derivatization	GC-MS	No	[88]
Baldi *et al.* 2021	CRC vs healthy	19 CRC, 16 healthy	Derivatization	GC-MS	No	[84]
Barberini *et al.* 2021	Pre vs post CRC	15 pre-surgery, 15 CRC post-surgery	Derivatization	GC-MS	No	[95]
Genua *et al.* 2021	CRC vs adenomas vs healthy	84 CRC, 66 adenomas, 63 controls	Derivatization	GC-MS	No	[45]

CRC: colorectal carcinoma, GC-MS: gas chromatography-mass spectrometry, SPME: solid phase microextraction RC: rectal carcinoma.

References

37 Cross, A.J.; Moore, S.C.; Boca, S.; Huang, W.-Y.; Xiong, X.; Stolzenberg-Solomon, R.; Sinha, R.; Sampson, J.N. A prospective study of serum metabolites and colorectal cancer risk. *Cancer* **2014**, *120*, 3049–3057. https://doi.org/10.1002/cncr.28799.

38 Crotti, S.; Agnoletto, E.; Cancemi, G.; Di Marco, V.; Traldi, P.; Pucciarelli, S.; Nitti, D.; Agostini, M. Altered plasma levels of decanoic acid in colorectal cancer as a new diagnostic biomarker. *Anal. Bioanal. Chem.* **2016**, *408*, 6321–6328. https://doi.org/10.1007/s00216-016-9743-1.

43 Di Giovanni, N.; Meuwis, M.A.; Louis, E.; Focant, J.F. Specificity of metabolic colorectal cancer biomarkers in serum through effect size. *Metabolomics* **2020**, *16*, 88. https://doi.org/10.1007/s11306-020-01707-w.

44 Farshidfar, F.; Weljie, A.M.; Kopciuk, K.A.; Hilsden, R.; McGregor, S.E.; Buie, W.D.; MacLean, A.; Vogel, H.J.; Bathe, O.F. A validated metabolomic signature for colorectal cancer: Exploration of the clinical value of metabolomics. *Br. J. Cancer* **2016**, *115*, 848–857. https://doi.org/10.1038/bjc.2016.243.

45 Genua, F.; Mirković, B.; Mullee, A.; Levy, M.; Gallagher, W.M.; Vodicka, P.; Hughes, D.J. Association of circulating short chain fatty acid levels with colorectal adenomas and colorectal cancer. *Clin. Nutr. ESPEN* **2021**, *46*, 297–304. https://doi.org/10.1016/j.clnesp.2021.09.740.

46 Hassan, H.A.; Ammar, N.M.; Serag, A.; Shaker, O.G.; El Gendy, A.N.; Abdel-Hamid, A.H.Z. Metabolomics driven analysis of obesity-linked colorectal cancer patients via GC-MS and chemometrics: A pilot study. *Microchem. J.* **2020**, *155*, 104742. https://doi.org/10.1016/j.microc.2020.104742.

48 Ikeda, A.; Nishiumi, S.; Shinohara, M.; Yoshie, T.; Hatano, N.; Okuno, T.; Bamba, T.; Fukusaki, E.; Takenawa, T.; Azuma, T.; et al. Serum metabolomics as a novel diagnostic approach for gastrointestinal cancer. *Biomed. Chromatogr.* **2012**, *26*, 548–558. https://doi.org/10.1002/bmc.1671.

53 Kim, S.; Yin, X.; Prodhan, M.A.I.; Zhang, X.; Zhong, Z.; Kato, I. Global plasma profiling for colorectal cancer-associated volatile organic compounds: A proof-of-principle study. *J. Chromatogr. Sci.* **2019**, *57*, 385–396. https://doi.org/10.1093/chromsci/bmz011.

56 Ma, Y.; Liu, W.; Peng, J.; Huang, L.; Zhang, P.; Zhao, X.; Cheng, Y.; Qin, H. A pilot study of gas chromatograph/mass spectrometry-based serum metabolic profiling of colorectal cancer after operation. *Mol. Biol. Rep.* **2010**, *37*, 1403–1411. https://doi.org/10.1007/s11033-009-9524-4.

57 Ma, Y.; Zhang, P.; Wang, F.; Liu, W.; Yang, J.; Qin, H. An integrated proteomics and metabolomics approach for defining oncofetal biomarkers in the colorectal cancer. *Ann. Surg.* **2012**, *255*, 720–730. https://doi.org/10.1097/SLA.0b013e31824a9a8b.

66 Nishiumi, S.; Kobayashi, T.; Ikeda, A.; Yoshie, T.; Kibi, M.; Izumi, Y.; Okuno, T.; Hayashi, N.; Kawano, S.; Takenawa, T.; et al. A novel serum metabolomics-based diagnostic approach for colorectal cancer. *PLoS ONE* **2012**, *7*, e40459. https://doi.org/10.1371/journal.pone.0040459.

67 Nishiumi, S.; Kobayashi, T.; Kawana, S.; Unno, Y.; Sakai, T.; Okamoto, K.; Yamada, Y.; Sudo, K.; Yamaji, T.; Saito, Y.; et al. Investigations in the possibility of early detection of colorectal cancer by gas chromatography/triple-quadrupole mass spectrometry. *Oncotarget* **2017**, *8*, 17115–17126. https://doi.org/10.18632/oncotarget.15081.

76 Qiu, Y.; Cai, G.; Su, M.; Chen, T.; Zheng, X.; Xu, Y.; Ni, Y.; Zhao, A.; Xu, L.X.; Cai, S.; et al. Serum metabolite profiling of human colorectal cancer using GC-TOFMS and UPLC-QTOFMS. *J. Proteome. Res.* **2009**, *8*, 4844–4850. https://doi.org/10.1021/pr9004162.

79 Shu, X.; Xiang, Y.-B.; Rothman, N.; Yu, D.; Li, H.-L.; Yang, G.; Cai, H.; Ma, X.; Lan, Q.; Gao, Y.-T.; et al. Prospective study of blood metabolites associated with colorectal cancer risk. *Int. J. Cancer* **2018**, *143*, 527–534. https://doi.org/10.1002/ijc.31341.

81 Tan, B.; Qiu, Y.; Zou, X.; Chen, T.; Xie, G.; Cheng, Y.; Dong, T.; Zhao, L.; Feng, B.; Hu, X.; et al. Metabonomics identifies serum metabolite markers of colorectal cancer. *J. Proteome Res.* **2013**, *12*, 3000–3009. https://doi.org/10.1021/pr400337b.

83 Uchiyama, K.; Yagi, N.; Mizushima, K.; Higashimura, Y.; Hirai, Y.; Okayama, T.; Yoshida, N.; Katada, K.; Handa, O.; Ishikawa, T.; et al. Serum metabolomics analysis for early detection of colorectal cancer. *J. Gastroenterol.* **2017**, *52*, 677–694. https://doi.org/10.1007/s00535-016-1261-6.

84 Baldi, S.; Menicatti, M.; Nannini, G.; Niccolai, E.; Russo, E.; Ricci, F.; Pallecchi, M.; Romano, F.; Pedone, M.; Poli, G.; et al. Free fatty acids signature in human intestinal disorders: Significant association between butyric acid and celiac disease. *Nutrients* **2021**, *13*, 742. https://doi.org/10.3390/nu13030742.

86 Wang, C.; Li, P.; Lian, A.; Sun, B.; Wang, X.; Guo, L.; Chi, C.; Liu, S.; Zhao, W.; Luo, S.; et al. Blood volatile compounds as biomarkers for colorectal cancer. *Cancer Biol. Ther.* **2014**, *15*, 200–206. https://doi.org/10.4161/cbt.26723.

88 Wu, J.; Wu, M.; Wu, Q. Identification of potential metabolite markers for colon cancer and rectal cancer using serum metabolomics. *J. Clin. Lab. Anal.* **2020**, *34*, e23333. https://doi.org/10.1002/jcla.23333.

91 Yuan, F.; Kim, S.; Yin, X.; Zhang, X.; Kato, I. Integrating two-dimensional gas and liquid chromatography-mass spectrometry for untargeted colorectal cancer metabolomics: A proof-of-principle study. *Metabolites* **2020**, *10*, 343. https://doi.org/10.3390/metabo10090343.

95 Barberini, L.; Restivo, A.; Noto, A.; Deidda, S.; Fattuoni, C.; Fanos, V.; Saba, L.; Zorcolo, L.; Mussap, M. A gas chromatography-mass spectrometry (GC-MS) metabolomic approach in human colorectal cancer (CRC): The emerging role of monosaccharides and amino acids. *Ann. Transl. Med.* **2019**, *7*, 727–727. https://doi.org/10.21037/atm.2019.12.34.

Table S5. Summary of studies analyzing VOCs in tissue for the detection of colorectal neoplasia compared to controls.

**Author**	**Comparison**	**Samples**	**Sampling method**	**Analysis method**	**Microbiota analysis**	**Reference**
Zimmerman *et al.* 2007	CRC vs healthy	N/A	SPME	GC-MS	No	[94]
Denkert *et al.* 2008	CRC vs healthy	27 CRC 18 healthy	Derivatization	GC-MS	No	[42]
Mal *et al.* 2009	CRC vs healthy	6 CRC, 6 healthy	Derivatization	GC-MS	No	[59]
Ong *et al.* 2010	CRC + AA vs healthy	26 CRC, 26 healthy tissue	Derivatization	GC-MS	No	[69]
Mal *et al.* 2012	CRC vs healthy	31 CRC, 31 healthy	Derivatization	GCxGC-MS	No	[58]
Nugent *et al.* 2014	Adenomas vs healthy	15 CRC 15 healthy	Derivatization	GC-MS	Yes	[68]
Qiu *et al.* 2014	CRC vs healthy	376 CRC	Derivatization	GC-MS	No	[77]
Brown *et al.* 2016	CRC vs healthy tissue	16 CRC vs 17 healthy	Derivatization	GC-MS	No	[32]
Krishnamurthy *et al.* 2017	CRC vs healthy	4 CRC, 4 healthy	Derivatization	gc-IRMS	No	[54]
Ning *et al.* 2017	CRC vs healthy	20 CRC, 20 healthy	Derivatization	GC-MS	No	[64]
Kibi *et al.* 2019	CRC vs healthy	10 CRC, 10 healthy	Derivatization	GC-MS	No	[50]
Liu *et al.* 2019	CRC vs healthy	6 CRC, 6 healthy	SPME	GC-MS	No	[55]
Arshad *et al.* 2019	CRC vs healthy	N/A	Syringe	GC-MS	No	[73]
Zhu *et al.* 2021	CRC vs healthy	48 CRC 48 healthy	Derivatization	GC-MS	No	[93]
Barberis *et al.* 2021	AA vs healthy	10 AA, 10 healthy	Derivatization	GCxGC-MS	No	[97]
Hou *et al.* 2021	CRC vs healthy	3 CRC,3 healthy	Derivatization	GC-MS	No	[47]
De Vietro *et al.* 2021	CRC vs healthy tissue	3 CRC 3 vs healthy	SPME	GC-MS	No	[39]

N/A: not available, CRC: colorectal carcinoma, AA: advanced adenoma, SPME: solid phase microextraction, GC-MS: gas chromatography-mass spectrometry,

References

32 Brown, D.G.; Rao, S.; Weir, T.L.; O'Malia, J.; Bazan, M.; Brown, R.J.; Ryan, E.P. Metabolomics and metabolic pathway networks from human colorectal cancers, adjacent mucosa, and stool. *Cancer Metab*. **2016**, *4*, 11. https://doi.org/10.1186/s40170-016-0151-y.

39 De Vietro, N.; Aresta, A.M.; Picciariello, A.; Rotelli, M.T.; Zambonin, C. Determination of VOCs in surgical resected tissues from colorectal cancer patients by solid phase microextraction coupled to gas chromatography–mass spectrometry. *Appl. Sci*. **2021**, *11*, 6910. https://doi.org/10.3390/app11156910.

42 Denkert, C.; Budczies, J.; Weichert, W.; Wohlgemuth, G.; Scholz, M.; Kind, T.; Niesporek, S.; Noske, A.; Buckendahl, A.; Dietel, M.; et al. Metabolite profiling of human colon carcinoma—Deregulation of TCA cycle and amino acid turnover. *Mol. Cancer* **2008**, *7*, 72. https://doi.org/10.1186/1476-4598-7-72.

47 Hou, X.; Hu, J.; Zhao, X.; Wei, Q.; Zhao, R.; Li, M.; Li, Q. Taurine Attenuates the Hypotaurine-Induced Progression of CRC via ERK/RSK Signaling. *Front. Cell Dev. Biol*. **2021**, *9*, 631163. https://doi.org/10.3389/fcell.2021.631163.

50 Kibi, M.; Nishiumi, S.; Kobayashi, T.; Kodama, Y.; Yoshida, M. GC/MS and LC/MS-based tissue metabolomic analysis detected increased levels of antioxidant metabolites in colorectal cancer. *Kobe J. Med. Sci*. **2019**, *65*, E19–E27.

54 Krishnamurthy, R.V.; Suryawanshi, Y.R.; Essani, K. Nitrogen isotopes provide clues to amino acid metabolism in human colorectal cancer cells. *Sci. Rep*. **2017**, *7*, 2562. https://doi.org/10.1038/s41598-017-02793-y.

55 Liu, M.; Li, Y.; Wang, G.; Guo, N.; Liu, D.; Li, D.; Guo, L.; Zheng, X.; Yu, K.; Yu, K.; et al. Release of volatile organic compounds (VOCs) from colorectal cancer cell line LS174T. *Anal. Biochem*. **2019**, *581*, 113340. https://doi.org/10.1016/j.ab.2019.06.011.

58 Mal, M.; Koh, P.K.; Cheah, P.Y.; Chan, E.C.Y. Metabotyping of human colorectal cancer using two-dimensional gas chromatography mass spectrometry. *Anal. Bioanal Chem*. **2012**, *403*, 483–493. https://doi.org/10.1007/s00216-012-5870-5.

59 Mal, M.; Koh, P.K.; Cheah, P.Y.; Chan, E.C.Y. Development and validation of a gas chromatography/mass spectrometry method for the metabolic profiling of human colon tissue. *Rapid Commun. Mass Spectrom*. **2009**, *23*, 487–494. https://doi.org/10.1002/rcm.3898.

64 Ning, W.; Li, H.; Meng, F.; Cheng, J.; Song, X.; Zhang, G.; Wang, W.; Wu, S.; Fang, J.; Ma, K.; et al. Identification of differential metabolic characteristics between tumor and normal tissue from colorectal cancer patients by gas chromatography–mass spectrometry. *Biomed. Chromatogr*. **2017**, *31*, e3999. https://doi.org/10.1002/bmc.3999.

68 Nugent, J.L.; McCoy, A.N.; Addamo, C.J.; Jia, W.; Sandler, R.S.; Keku, T.O. Altered tissue metabolites correlate with microbial dysbiosis in colorectal adenomas. *J. Proteome. Res*. **2014**, *13*, 1921–1929. https://doi.org/10.1021/pr4009783.

69 Ong, E.S.; Zou, L.; Li, S.; Cheah, P.Y.; Eu, K.W.; Ong, C.N. Metabolic profiling in colorectal cancer reveals signature metabolic shifts during tumorigenesis. *Mol. Cell. Proteom.* **2010**. https://doi.org/10.1074/mcp.m900551-mcp200.

73 Arshad, A.Z.; Munajat, Y.; Ghoshal, S.K.; Jamal, R.; Johdi, N.A.; Ibrahim, R.K.R.; Zulkhairi, H. Volatolomics combined terahertz time domain spectral analyses of colon cancer in vitro. *J. Teknol*. **2019**, *81*, 105–112. https://doi.org/10.11113/jt.v81.13310.

77 Qiu, Y.; Cai, G.; Zhou, B.; Li, D.; Zhao, A.; Xie, G.; Li, H.; Cai, S.; Xie, D.; Huang, C.; et al. A distinct metabolic signature of human colorectal cancer with prognostic potential. *Clin. Cancer Res*. **2014**, *20*, 2136–2146. https://doi.org/10.1158/1078-0432.CCR-13-1939.

93 Zhu, G.; Wang, Y.; Wang, W.; Shang, F.; Pei, B.; Zhao, Y.; Kong, D.; Fan, Z. Untargeted GC-MS-Based Metabolomics for Early Detection of Colorectal Cancer. *Front. Oncol*. **2021**, *11*, 729512. https://doi.org/10.3389/fonc.2021.729512.

94 Zimmermann, D.; Hartmann, M.; Moyer, M.P.; Nolte, J.; Baumbach, J.I. Determination of volatile products of human colon cell line metabolism by GC/MS analysis. *Metabolomics* **2007**, *3*, 13–17. https://doi.org/10.1007/s11306-006-0038-y.

97 Barberis, E.; Joseph, S.; Amede, E.; Clavenna, M.G.; La Vecchia, M.; Sculco, M.; Aspesi, A.; Occhipinti, P.; Robotti, E.; Boldorini, R.; et al. A new method for investigating microbiota-produced small molecules in adenomatous polyps. *Anal. Chim. Acta*. **2021**, *1179*, 338841. https://doi.org/10.1016/j.aca.2021.338841.
