# Supplementary material for: Systematic Review: Contribution of the Gut Microbiome to the Volatile Metabolic Fingerprint of Colorectal Neoplasia
Source: Metabolites. 2022 Dec 30;13(1):55. doi: 10.3390/metabo13010055 (PMC9865897; doi:10.3390/metabo13010055)
Supplement: Supplementary file 1 [file metabolites-13-00055-s001.zip › metabolites-2087034-supplementary/Supplementary_Materials_S5_Included_Microbiota_Studies.pdf]

Supplementary Materials – Included studies on gut microbiota associated with colorectal neoplasia

Huybrechts *et al.* 2020 CRC vs. controls.

**Taxa**	**Name**	**Biosample**	**CRC enriched/depleted**
Phylum			
	Fusobacteriota	feces, tissue	Enriched
Family			
	Fusobacteriaceae	feces, tissue	Enriched
	Lachnospiraceae	feces, tissue	Depleted
	Peptostreptococcaceae	feces	Enriched
	Porhyromonadaceae	feces	Enriched
	Rimunococcaceae	feces, tissue	Depleted
Genus			
	Acinetobacter	feces	Depleted
	Atopobium	feces, tissue	Enriched
	Bifidobacterium	feces, tissue	Depleted
	Blautia	feces, tissue	Depleted
	Desulfovibrio	feces	Enriched
	Fusobacterium	feces, tissue	Enriched
	Gemella	feces, tissue	Enriched
	Leptotrichia	feces, tissue, saliva	Enriched
	Odoribacter	feces	Enriched
Species	*Oscillibacter*	feces	Enriched
	Parvimonas	feces	Enriched
	Peptostreptococcus	feces, tissue	Enriched
	Porphyromonas	feces, tissue	Enriched
	Prevotella	feces	Enriched
	Staphylococcus	feces	Depleted
	Bacteroides fragilis	feces, tissue	Enriched
	Blautia coccoides	fecees	Enriched
	Escherichia coli	feces, tissue	Enriched
	Fusobacterium nucleatum	feces, tissue	Enriched
	Parvimonas micra	feces	Enriched
	Peptostreptococcus stomatis	feces	Enriched
	Porphyromonas asaccharolytica	feces	Enriched
	Prevotella intermedia	feces, saliva	Enriched

Amitay *et al. 2018* CRC or adenomas vs healthy controls

**Taxa**	**Name**	**Biosample**	**CRC enriched/depleted**
Phylum			
	Fusobacteriota	feces	Enriched
	Bacillota (Firmicutes)	feces	Depleted
Family			
	Clostridiales	feces	Depleted
	Lachnospiraceae	feces	Mixed
	Porphyromonadaceae	feces	Enriched (+ enriched in adenoma)
Genus			
	Roseburia	feces	Depleted
	Eubacterium	feces	Depleted
	Parvimonas	feces	Enriched
	Peptostreptococcus	feces	Enriched
	Ruminococcus	feces	Depleted
	Selenomonas	feces	Enriched
	Streptococcus	feces	Mixed in adenoma only
	Fusobacterium	feces	Enriched (+ enriched in adenoma)
	Leptotrichia	feces	Enriched
	Porphyromonas	feces	Enriched
	Bacteroides	feces	Mixed
	Atopobium	feces	Enriched
	Bifidobacterium	feces	Depleted
	Bilophila	feces	Enriched
Species	*Roseburia intestinalis*	feces	Depleted
	Eubacterium ventriosum	feces	Depleted
	Parvimonas micra	feces	Enriched
	Peptostreptococcus stomatis	feces	Mixed
	Fusobacterium nucleatum	feces	Enriched (+ enriched in adenoma)

Borges-Canha *et al. 2015* CRC or adenomas vs controls. Animal studies excluded.

Taxa	Name	Biosample	CRC enriched/depleted
Phylum			
	Actinomycetota (subclass Coriobacteridae)	tissue	Enriched
	Pseudomonadota (Proteobacteria)	tissue	Enriched (+ enriched in adenoma)
Family			
	Eubacteriaceae	feces	Depleted
	Staphylococcaceae	feces	Enriched
	Enterobacteriaceae	feces, tissue	Mixed (+ enriched in adenoma)
	Campylobacteraceae	feces	Enriched
Genus			
	Fusobacterium	feces, tissue	Enriched (+ enriched in adenoma)
	Bacteroides	feces, tissue	Enriched (+ mixed in adenoma)
	Prevotella	feces, tissue	Enriched
	Porphyromonas	feces	Enriched
	Dysgonomonas	feces	Enriched
	Bifidobacterium	feces, tissue	Depleted
	Slackia	tissue	Enriched
	Collinsella	tissue	Enriched
	Lactobacillus	feces	Depleted
	Roseburia	feces	Depleted
Species	*Fusobacterium nucleatum*	tissue	Enriched
	Faecalibacterium prausnitzii	feces, tissue	Depleted
	Akkermansia muciniphila	feces	Enriched
	Faecalibacterium spp	feces	Enriched in adenoma only

Liu ***et al. 2016*** CRC vs healthy controls

**Taxa**	**Name**	**Biosample**	**CRC enriched/depleted**
Family			
	Enterobacteriaceae	feces	Enriched
Genus			
	Bifidobacterium	feces	Depleted
	Fusobacterium	feces	Enriched
Species			
	Faecalibacterium prausnitzii	feces	Depleted
	Enterococcus faecalis	feces	Enriched
	Enterococcus faecium	feces	Enriched

Aprile *et al. 2021* Adenomas vs controls.

**Taxa**	**Name**	**Biosample**	**Adenoma enriched/depleted**
Phylum			
	Pseudomonadota (Proteobacteria)	tissue	Enriched
	Bacillota (Firmicutes)	tissue	Depleted
Genus	*Pseudomonas*	tissue	Enriched
	Helicobacter	tissue	Enriched
	Acinetobacter	tissue	Enriched
	Fusobacteria	feces	Enriched
	Porphyromonas	feces	Enriched
	Lactobacillus	feces	Depleted
	Roseburia	feces	Depleted
	Clostridium	feces	Depleted
	Bifidobacterium	feces, tissue	Depleted
	Enterococcus	feces	Enriched
	Streptococcus	feces	Enriched
	Faecalibacterium	tissue	Depleted
	Romboutsia	tissue	Depleted
Species			
	Fusobacterium nucleatum	feces	Enriched
	Fusobacterium varium	feces	Enriched
	Fusobacteriuim mortiferum	tissue	Enriched
	Enterococcus faecalis	feces	Enriched
	Streptococcis bovis	feces	Enriched
	Bacteroides fragilis	feces	Enriched
	Faecalibacterium prausnitzii	tissue	Depleted
	Bifidobacterium pseudocatenalulatum	tissue	Depleted
	Bacteroides massiliensis	feces	Enriched
	Bacteroides dorei	feces	Enriched
	Escherichia coli	tissue	Enriched
